# Mapping the landscape of anaemia research in pregnant and postpartum women: a bibliometric analysis & topic modelling study

**DOI:** 10.3389/fgwh.2026.1851276

**Published:** 2026-07-24

**Authors:** Olalekan A. Uthman, Tabassum Firoz, María Barreix, Lisa M. Rogers

**Affiliations:** 1 Warwick Centre for Global Health Research, Applied Health, Warwick Medical School, The University of Warwick, Coventry, United Kingdom; 2 Department of Medicine, Yale School of Medicine and Bridgeport Hospital, Yale New Haven Health, Bridgeport, CT, United States; 3 UNDP/UNFPA/UNICEF/WHO/World Bank Special Programme of Research, Development and Research Training in Human Reproduction (HRP), Department of Sexual and Reproductive Health and Research, World Health Organization, Geneva, Switzerland; 4 Department of Nutrition and Food Safety, World Health Organization (WHO), Geneva, Switzerland

**Keywords:** maternal anemia, maternal nutrition, bibliometric analysis, evidence mapping, pregnancy

## Abstract

**Background:**

Maternal anaemia affects 35.5% of pregnant women globally and represents a persistent public health challenge with significant maternal and neonatal health consequences. Despite substantial research investment, progress towards anaemia reduction remains limited, suggesting potential misalignment between research priorities and translation to guidance and/or implementation of recommended interventions. Understanding the knowledge structure and thematic evolution of maternal anaemia research is essential for identifying gaps and informing future research strategies and guideline development.

**Objectives:**

This study aimed to map the knowledge structure and evolution of research on anaemia in pregnant and postpartum women from 2000 to 2024, analyse global collaboration networks and geographic distribution, and identify emerging themes and research gaps using bibliometric analysis and topic modelling.

**Methods:**

A comprehensive bibliometric analysis was conducted using Web of Science Core Collection database, encompassing 1,007 articles published between 2000 and 2024. Standard bibliometric indicators were calculated using VOSviewer and CiteSpace software. Latent Dirichlet Allocation topic modelling identified thematic clusters. Linear regression analysis examined temporal trends and geographic patterns.

**Results:**

Publication output demonstrated exponential growth (R^2^ = 0.9054), increasing from 12 articles in 2000 to 110 in 2024. Research output was concentrated amongst authors from high-income countries, with 68.5% of publications having first or corresponding authors based in high-income countries. Geographic disparities revealed substantial regional research gaps, particularly in Sub-Saharan Africa and South Asia. Topic modelling revealed nine clusters dominated by health outcomes research (77.4%), with less attention to management (21.6%), prevention (20.8%) and diagnosis (6.3%).

**Conclusions:**

This bibliometric analysis reveals substantial growth in maternal anaemia research over 25 years, with publications increasing nearly ten-fold. However, the research landscape shows thematic imbalances. Sustained attention is needed on prevention and diagnosis as well as the integrated delivery of anaemia interventions within multisectoral approaches to generate evidence and address remaining knowledge gaps.

## Background

Maternal anaemia remains one of the most persistent global public health challenges affecting approximately 35.5% of pregnant women worldwide in 2023. The prevalence of anaemia in this population group is as high as 52% in low- and middle-income countries, particularly in Sub-Saharan Africa and South Asia (1). Despite decades of intervention efforts, global progress towards anaemia reduction has remained stagnant (2), reflecting the complex interactions between nutritional deficiencies, obstetric causes, chronic and infectious diseases, and the broader socio-economic context (3).

Maternal anaemia can range from mild to severe and increase the risk of adverse health, development and economic outcomes. The health consequences include an increased risk of maternal and neonatal mortality and morbidity, such as postpartum haemorrhage, pre-eclampsia, low birth weight, along with intergenerational transmission of poor health outcomes (4). Both direct and indirect causes of maternal death can be due to anaemia. The most recent systematic analysis of the global causes of maternal death shows that hemorrhage, for which anaemia may be a risk factor, remains the leading cause of maternal deaths globally (5). The analysis also shows that there has been no substantial change in the proportion of maternal deaths from indirect causes since the two previous WHO analyses in 2006 and 2014.

Previous reviews of anaemia research have highlighted fragmentation and misalignment with public health priorities. Research attention has predominantly focused on nutritional supplementation strategies, particularly iron and folic acid interventions for the prevention of anaemia, whilst implementation science and health systems research, as well as research addressing the management of anaemia and its underlying causes, remain underrepresented (6, 7). Within the maternal health continuum, the postpartum period has received disproportionately limited research attention, with only three of 27 systematic reviews addressing postpartum anaemia management (6, 7).

In 2023, WHO launched *Accelerating anaemia reduction: a comprehensive framework for action*, advocating for coordinated action across systems and emphasizing a broad approach to diagnosis, prevention and management (3). This includes addressing all main causes of anaemia and the broader social inequities related to education, poverty, food insecurity and lack of access to family planning, health and nutrition services and clean water, sanitation and hygiene.

The complex causes of maternal anaemia and the heterogenous research landscape necessitate comprehensive evidence mapping approaches to characterise knowledge structures, identify research gaps, and inform research priorities for guideline development. Bibliometric analysis provides powerful methodological approaches for synthesising large bodies of research, enabling identification of collaboration patterns, thematic evolution, and geographic distribution of research activity (8). Topic modelling techniques, particularly Latent Dirichlet Allocation (LDA), offer sophisticated approaches for identifying latent thematic structures within extensive literature collections, facilitating objective assessment of research priorities and trends (9).

Previous bibliometric analyses in related maternal health domains have demonstrated the utility of these approaches for identifying research inequities, collaboration patterns, and emerging research frontiers (10). However, no comprehensive bibliometric analysis has specifically examined the landscape of anaemia research in pregnant and postpartum women using contemporary computational methods integrated with policy-relevant frameworks.

The aim of this study is to address these knowledge gaps through comprehensive bibliometric analysis and topic modelling of anaemia research in pregnant and postpartum women by: (1) mapping the knowledge structure and evolution of research on anaemia in pregnant and postpartum women over the past 25 years; (2) analysing geographic distribution and global collaboration networks of research activity to identify patterns of research concentration and underrepresentation; and (3) uncovering emerging research themes and declining areas through temporal trend analysis and topic modelling.

## Methods

### Study design

**Figure 1:**
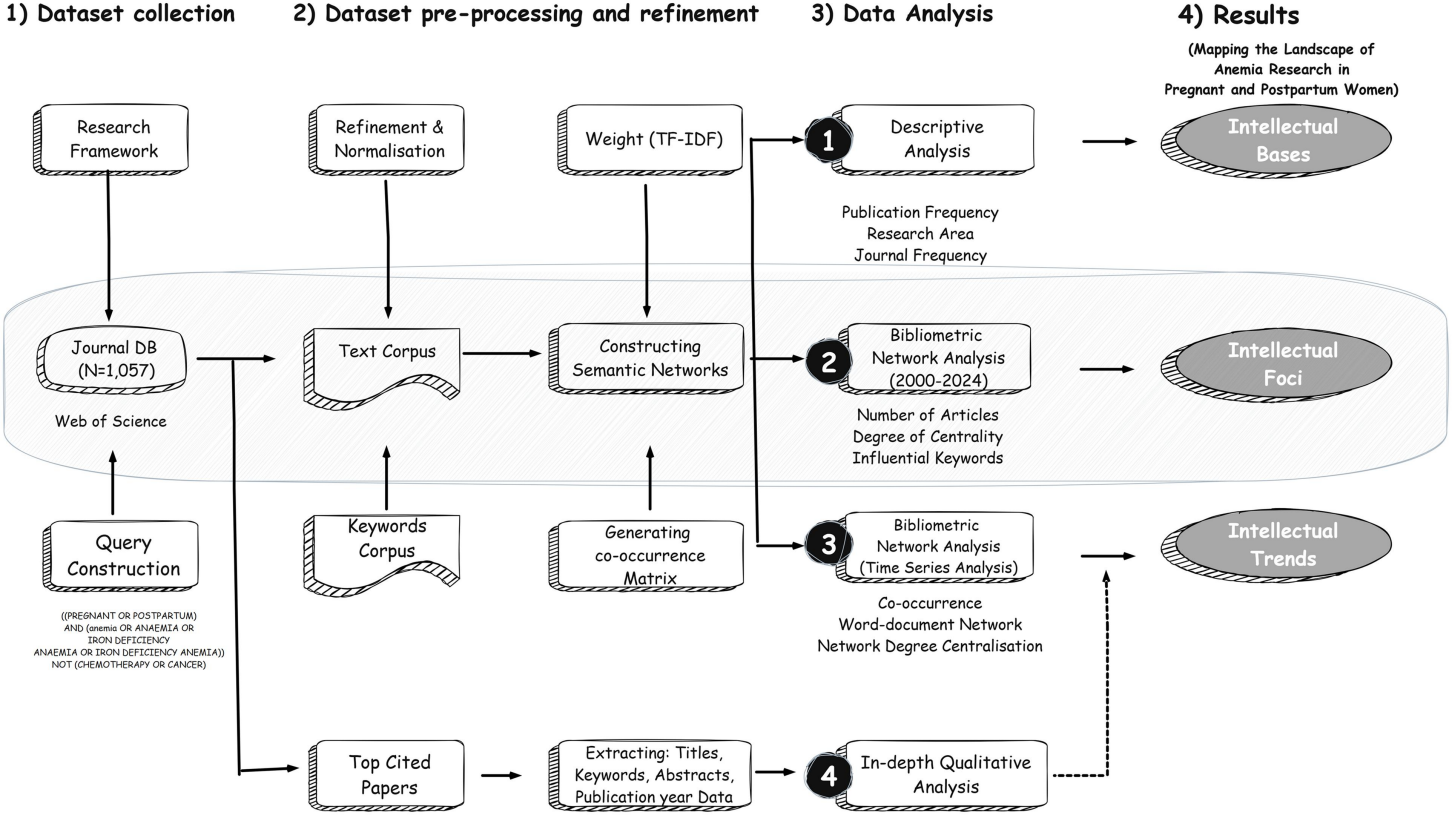
Overview of the methodology.

This research employed a comprehensive bibliometric analysis and topic modelling approach to systematically map the intellectual landscape of anaemia research in pregnant and postpartum women from 2000 to 2024 (Figure 1). The study integrated quantitative bibliometric indicators with qualitative content analysis through advanced computational techniques, including network analysis, topic modelling, and temporal trend assessment. This mixed-methods design enabled examination of research productivity patterns, collaboration networks, thematic evolution, and geographic distribution. Figure 1 provides an overview of the methodology. Each step is discussed in detail in Supplementary Appendix S1.

The Web of Science Core Collection was selected as the primary bibliographic source for three reasons: its comprehensive coverage of high-impact peer-reviewed journals across biomedical, public health and clinical disciplines; the availability of structured metadata fields (including author affiliations, funding bodies and citation counts) required for network and temporal analysis; and its native compatibility with VOSviewer and CiteSpace, the software used for co-authorship and keyword co-occurrence analysis. A recognised limitation of this choice is that Web of Science exhibits known under-coverage of journals from low- and middle-income countries and of publications in languages other than English (17). Whilst no language restriction was applied to the search strategy, English-language publications are disproportionately indexed, meaning that relevant research published in local languages or through regional platforms such as LILACS and African Journals Online may not be captured. These factors may have contributed to the geographic concentration of research output in high-income countries observed in our findings and are acknowledged in the Limitations section.

We additionally performed manual classification of all included studies against general categories for guideline development to serve as a reference standard (“ground truth”) for evaluating the distribution of machine-generated topics. Two reviewers independently assigned categories with disagreements resolved by consensus.

### Interactive dashboard development

A comprehensive interactive dashboard was developed and deployed to facilitate detailed exploration of the bibliometric analysis findings (https://maternalanemia.streamlit.app/) (Supplementary Appendix S1). This web-based platform provides stakeholders with dynamic access to the complete analytical dataset through multiple interconnected visualisation modules designed for different user needs and expertise levels. The dashboard comprises several key analytical sections enabling comprehensive data exploration. Advanced analytical tools include an Authors Discovery module for identifying influential researchers and collaboration patterns, and a Topics Discovery section providing detailed thematic analysis with interactive topic model exploration.

## Results

**Figure 2:**
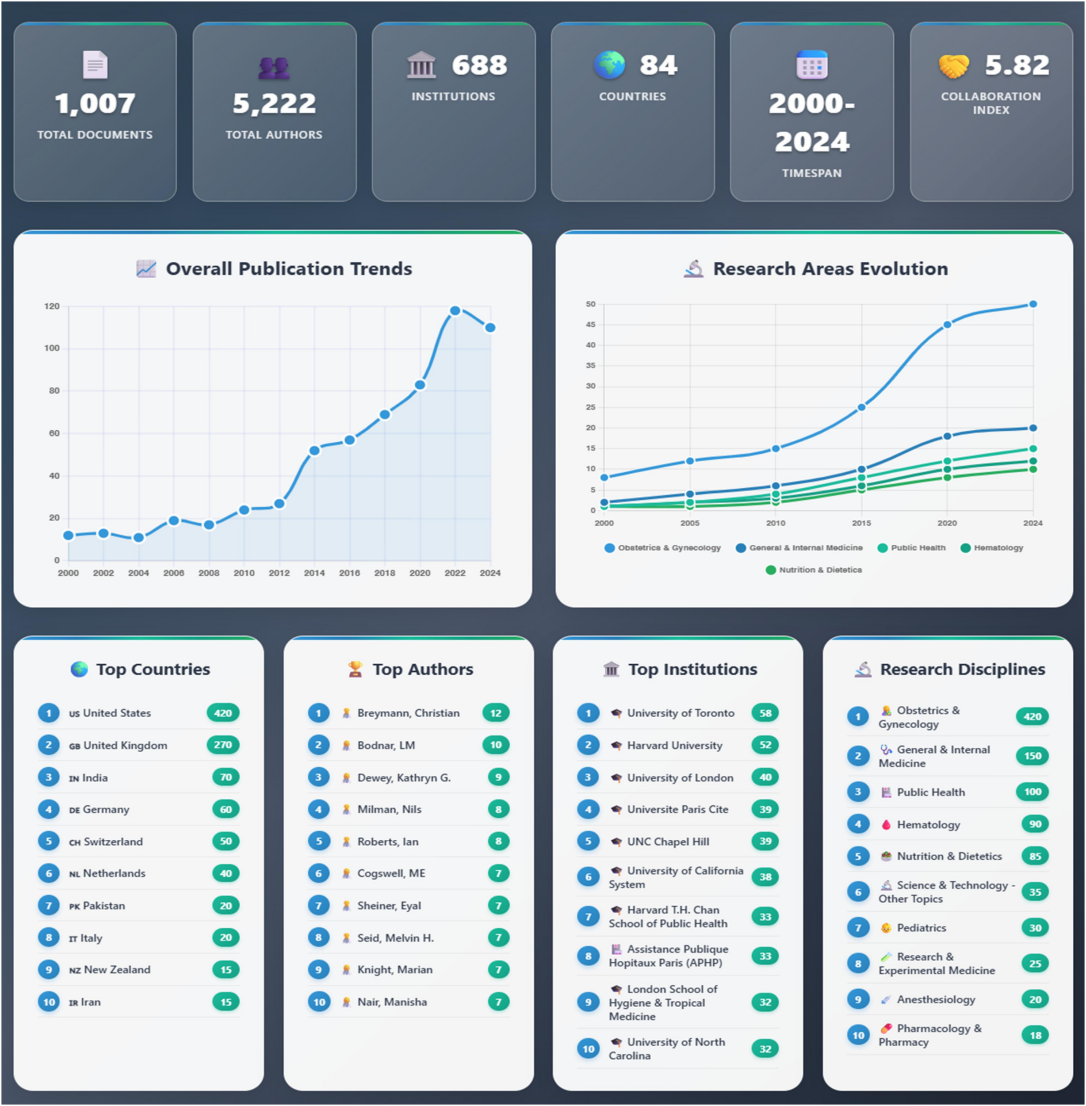
Overview of bibliometric analysis of anaemia research in pregnant and postpartum women (2000-2024).panel A: research output metrics; panel B: publication and disciplinary trends; panel C: Top contributors by country, author, institution, and research discipline.

The bibliometric analysis encompassed 1,007 articles on anaemia in pregnant and postpartum women published between 2000 and 2024 (Figure 2, Supplementary eFigure S1 and eFigure S2, Appendix S1). The dataset comprised 814 research articles (80.8%), 110 reviews (10.9%), 51 meeting abstracts (5.1%), 18 proceedings papers (1.8%), and smaller proportions of other article types including editorials, corrections, and news items. Of the 1,007 included articles, 461 (45.8%) primarily addressed anaemia in pregnancy, 411 (40.8%) primarily addressed the postpartum period, covering postpartum haemorrhage, post-caesarean haemoglobin recovery and puerperal anaemia, and 122 (12.1%) addressed both phases explicitly.

### Publication trends & temporal publication patterns

Publications originated from 84 countries across 688 institutions and were disseminated through 363 distinct sources (mainly academic journals). Annual publication output exhibited a marked exponential growth trajectory over the 25-year study period, with an R^2^ value of 0.9054 indicating strong model fit (Figure 2). Publications increased from 12 in 2000 to a peak of 110 in 2024, representing a nearly ten-fold increase. The trend demonstrated acceleration from 2012 onwards, with annual output rising from 35 publications in 2012 to sustained levels above 50 publications from 2015. Average annual publication output across the entire timespan was 40.28 articles per year.

### Terminology landscape and keyword evolution

**Figure 3:**
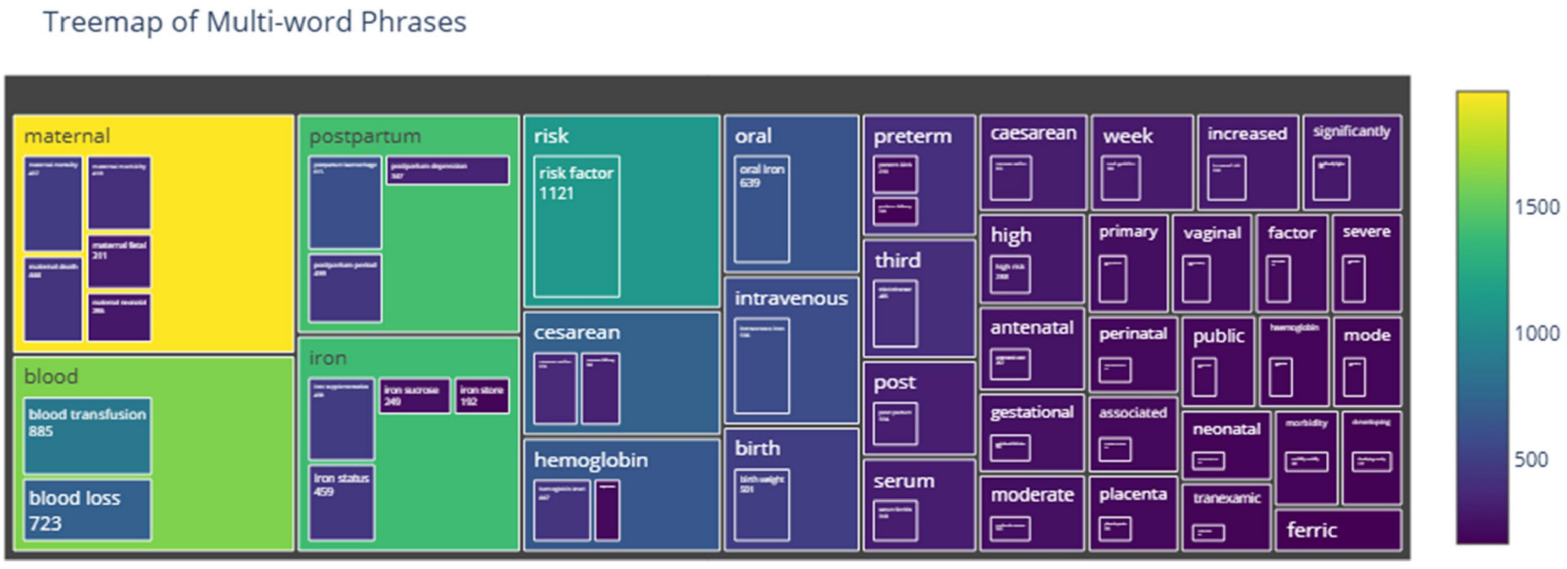
Terminology landscape.

The most frequently occurring multi-word phrases demonstrated clear thematic clustering around key research domains (Figure 3). Maternal-related terms dominated the largest segment, encompassing phrases such as “maternal mortality,” “maternal health,” and “maternal outcomes.” The postpartum domain represented another substantial cluster, including terms like “postpartum haemorrhage” and “postpartum depression.” Pregnancy-related terminology was represented through phrases including “preterm birth,” “caesarean section,” and gestational periods (“third trimester”). Haematological parameters constituted another cluster, featuring “haemoglobin levels,” “blood loss,” and “blood transfusion.” The frequency distribution, indicated by colour intensity, showed maternal-related phrases achieving the highest occurrence rates, followed by clinical intervention and diagnostic terminology (Figure 3).

**Figure 4:**
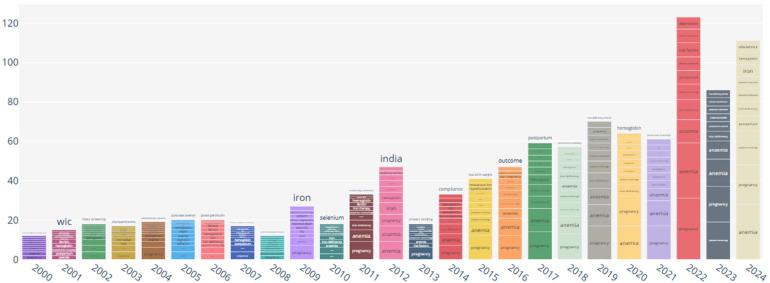
Evolution of keywords.

The keyword evolution analysis spanning 2000–2024 demonstrated dynamic shifts in research terminology and focus areas (Figure 4). Early years (2000–2003) were characterised by fundamental terminology including “anaemia,” “pregnancy,” and basic haematological markers. The period from 2004 to 2007 showed expansion into “iron deficiency” and “folate” supplementation research. A notable transition occurred from 2008 to 2010, with the emergence of “iron supplementation” as a prominent keyword, alongside increased attention to “outcomes” and “percentage of articles “ studies. The period 2011–2013 marked the introduction of more sophisticated clinical terminology, including specific treatment modalities and “India” as a geographical focus, reflecting increased research activity in high-burden regions.

The years 2014–2016 demonstrated diversification into “pregnancy outcomes,” “anaemia prevalence” and “prevention” strategies. A significant evolution occurred in 2017–2019, with the emergence of “depression,” “gestational diabetes,” and “quality of life” keywords, indicating research expansion beyond traditional haematological parameters into broader maternal health and wellbeing outcomes.

The most recent period (2020–2024) revealed further specialisation with keywords including “systematic review,” “meta-analysis,” “randomised controlled trial,” and “machine learning,” suggesting methodological advancement and evidence synthesis approaches. Geographic diversification continued with keywords representing various regions and populations, whilst clinical terminology evolved to include specific biomarkers and advanced therapeutic approaches.

### Research discipline distribution and trends

Research discipline analysis revealed the multidisciplinary nature of maternal anaemia research with clear domain predominance. Obstetrics and gynaecology represented the largest disciplinary contribution with 420 publications (41.7%), confirming the clinical focus on maternal health. General and internal medicine contributed 150 publications (14.9%), whilst public health accounted for 100 publications (9.9%). Haematology generated 90 publications (8.9%), and nutrition and dietetics contributed 85 publications (8.4%). Other disciplines included science and technology (3.5%), paediatrics (3.0%), research and experimental medicine (2.5%), anaesthesiology (2.0%), and pharmacology and pharmacy (1.8%). The research discipline trends over time demonstrated dynamic shifts in disciplinary engagement from 2000 to 2024. Obstetrics and gynaecology showed consistent growth throughout the period, with notable acceleration from 2015 onwards, reaching peak output exceeding 50 publications annually by 2024. General and internal medicine displayed moderate but steady growth, whilst public health contributions increased markedly after 2010.

### Geographic distribution and country networks

Country-level analysis revealed significant geographic concentration in maternal anaemia research productivity (Supplementary eFigure S3). The first authors or corresponding authors from the United States dominated with 420 publications (41.7%), followed by the United Kingdom with 270 publications (26.8%). First authors or corresponding authors from India contributed 70 publications (7.0%), representing the leading contribution from low- and middle-income countries.

The country collaboration network visualisation displayed extensive global research partnerships with notable regional clustering patterns. The network showed the United States and the United Kingdom as central nodes with multiple international connections. Strong collaborative relationships were evident between high-income countries, particularly among Anglo-American and European research partnerships. Lower- and middle-income countries, including India, Pakistan, and various African nations, demonstrated connections primarily with high-income country partners rather than substantial South-South collaboration.

### Regional topic focus analysis

**Figure 5:**
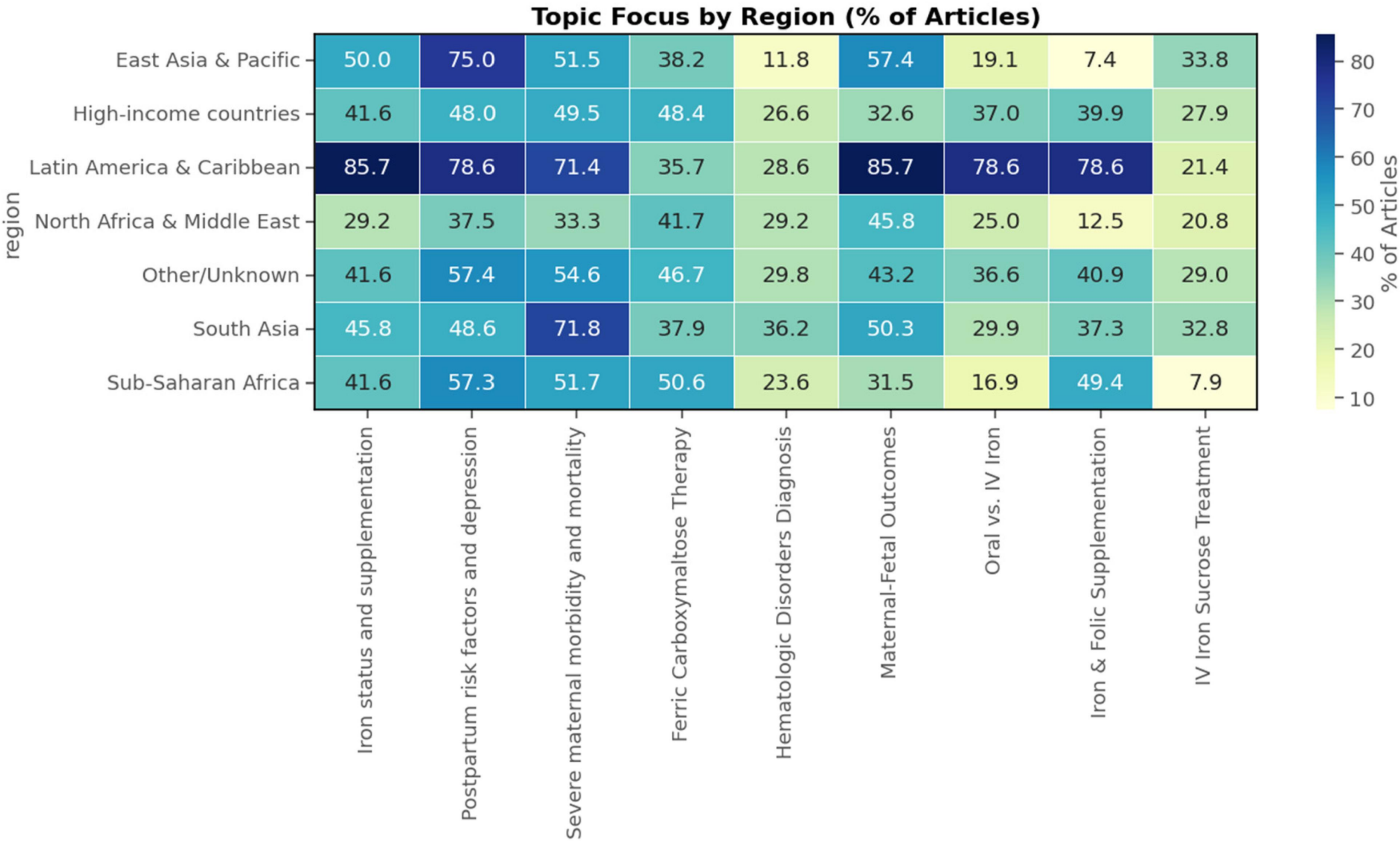
Topic focus by region (% of articles).

Geographic analysis of topic distribution across seven world regions revealed substantial variation in research priorities and thematic emphasis (Figure 5). The heatmap analysis demonstrated marked disparities in regional research focus, with topic percentage of articles ranging from 7.4% to 85.7% across different region-topic combinations, indicating pronounced geographic specialisation and potential research gaps. More detailed analysis can be found in Supplementary Appendix S3.

### Nine-cluster thematic structure

The topic modelling of the maternal anaemia literature corpus revealed nine distinct thematic clusters (Table 1 and Supplementary Appendix S4). The analysis grouped four primary research domains: prevention (20.8% of articles), diagnosis (6.3%), management (21.6%), and health outcomes (77.4%), with some articles spanning multiple categories. Comparison of the four categories relevant to guideline development revealed notable distributional differences between computationally derived clusters and manual classification (Supplementary Appendix S5). While the clustering identified health outcomes as the dominant research focus (77.4%), manual classification showed more balanced distribution across risk factors (37.7%), outcomes (31.2%), and treatment (28.8%), with prevention representing only 8.1% compared to the computational clustering's 20.8%.

**Table 1:** Topic classification and keywords .

Cluster	Topic keywords	Research Summary	Number of publications (%)
	Prevention		
#1: Iron status and supplementation	Iron status, concentration, status, infant, serum ferritin, vitamin, ferritin, serum, supplement	Nutritional interventions, biomarker assessment, impact of maternal iron deficiency on cognitive health	150 (14.9)
#8: Iron and folic acid supplementation	Iron supplementation, supplementation, factor associated, weight gain, acid, severe anaemia, malaria, postpartum period, folic	Anaemia prevention strategies particularly in low-income populations and HIV-infected pregnant women, supplementation efficacy, malaria prophylaxis, policy implications of universal iron supplementation programmes and intervention strategies for improving supplementation coverage in resource-limited settings.	59 (5.9)
	Diagnosis		
#5: Thrombocytopenia and postpartum complications	Antenatal care, puerperal sepsis, family planning, health facility, management, thalassemia, knowledge, postpartum period, thrombotic thrombocytopenic	Awareness gaps regarding anaemia signs in pregnancy, and management, diagnostic awareness of haematological disorders during pregnancy such as atypical haemolytic uraemic syndrome and thrombotic thrombocytopenic purpura	63 (6.3)
	Management		
#4: Ferric carboxymaltose and anemia management	Trial, ferric carboxymaltose, evidence, quality evidence, blood transfusion, postpartum haemorrhage, primary outcome, quality, blood loss	Evidence-based clinical management using intravenous ferric carboxymaltose, blood transfusion reduction, quality evidence assessment, optimal iron delivery methods, comparative effectiveness against standard medical care, and evidence-based guidelines for postpartum anaemia management.	87 (8.6)
#7: Intravenous vs. oral iron therapy	Oral iron, intravenous iron, post-partum, twin pregnancy, oral, intravenous, twin, blood transfusion, post	Clinical impacts of iron deficiency anaemia, therapeutic modalities for postpartum anaemia, comparative effectiveness studies of intravenous and oral iron, standardised treatment protocols, and implementation strategies.	71 (7.1)
#9: Iron sucrose treatment efficacy	Third trimester, iron sucrose, trimester, intravenous iron, sucrose, intravenous, oral, oral iron, third	Evaluation of intravenous iron sucrose for third trimester and postpartum anaemia, effectiveness of erythropoietin adjuvant therapy combined with iron sucrose, comparative analyses with oral iron supplementation, and	59 (5.9)
	Health Outcomes		
#2: Postpartum risk factors and depression	Risk factor, blood loss, postpartum depression, depression, caesarean section, loss, prevalence, caesarean delivery, tranexamic acid	Prediction models for postpartum haemorrhage, factors contributing to postpartum depression in various populations, and cell salvage impacts on postpartum anaemia following caesarean delivery.	250 (24.8)
Cluster #3: Severe maternal morbidity and mortality	Blood transfusion, maternal death, maternal morbidity, maternal mortality, postpartum haemorrhage, death, caesarean section, obstetric, morbidity	Blood transfusion requirements, and caesarean section complications, heart disease-related maternal deaths, severe obstetric morbidity near-miss maternal morbidity in women with anaemia	326 (32.4)
#6: Maternal-fetal outcomes and complications	Maternal fetal, birth weight, fetal, hemoglobin level, neonatal, gestational, weight, apgar score, platelet count	Thyroid dysfunction on maternal and fetal outcomes in women with anaemia, first-trimester haemoglobin relationships with pregnancy complications.	203 (20.2)

### Trends in research areas

#### Rapidly emerging topics

Two topics demonstrated statistically significant increasing trends with the steepest positive slopes. Ferric carboxymaltose therapy exhibited the most pronounced growth trajectory (slope = + 0.156, R^2^ = 0.186, *p* = 0.027), representing a 134.9% increase between early (2000-2005) and recent (2020-2024) periods. This cluster showed consistent upward progression from approximately 9% of articles in 2000 to peaks exceeding 15% by 2024. Oral vs. intravenous iron demonstrated the second-highest growth rate (slope = + 0.125, R^2^ = 0.129), reflecting increased comparative effectiveness research between therapeutic modalities.

#### Sustained growth areas

Severe maternal morbidity and maternal mortality maintained steady growth throughout the observation period (slope = + 0.085, R^2^ = 0.058, *p* = 0.246), with the percentage of articles increasing from approximately 12% in 2000 to consistent levels above 15% after 2015. Diagnosis of haematologic disorders showed moderate positive trends (slope = + 0.026, R^2^ = 0.028, *p* = 0.423), indicating gradual but sustained research expansion in diagnostic approaches.

#### Significantly declining topics

Iron status and supplementation demonstrated the most pronounced decline among all topics (slope=−0.267, R^2^ = 0.287, *p* = 0.006), showing statistically significant reductions from peaks of approximately 19% in early 2000s to below 10% by 2024. This represented a 24.9% decrease between comparative periods. Maternal-fetal outcomes showed moderate decline (slope=−0.015, R^2^ = 0.015, *p* = 0.555), with the percentage of articles decreasing from approximately 15% to below 10% over the study period.

#### Gradual decline

Iron and folic acid supplementation exhibited a declining trend (slope=−0.048, R^2^ = 0.048, *p* = 0.292), demonstrating reduced research focus on traditional supplementation approaches, decreasing from peaks above 15% to approximately 10% of articles. This decline reflected a 15.3% reduction in research attention over the comparative periods.

### The evolving landscape of maternal anaemia research funding

A total of 360 studies reported funding. There has been a remarkable 390% growth in the number of funded publications when comparing the period 2014–2024 to 2003–2013, indicating a growing recognition of the importance of this issue. The most frequently cited source was the Bill & Melinda Gates Foundation (48, 13.3%), followed by the U.S. National Institutes of Health (NIH) (25, 6.9%), the UK Medical Research Council (MRC), UK Research & Innovation (UKRI) (16, 4.4%), and the National Institute for Health and Care Research (NIHR) (10, 2.8%). Vifor Pharma and the Wellcome Trust were each acknowledged 9 times (2.5% each), the National Natural Science Foundation of China 8 times (2.2%), and the United States Agency for International Development 5 times (1.4%). A significant portion of publications are funded by multiple sources, with an average of 2.2 funders per publication. The network visualization reveals the key players in the co-funding landscape. The Bill & Melinda Gates Foundation emerges as a central hub, frequently collaborating with a wide range of other funders. The distribution of publications across clusters is uneven, with “Cluster 2: Postpartum risk factors and depression” and “Cluster 3: Severe maternal morbidity and mortality” receiving the most attention from funders. The funding portfolio of each cluster is unique. For example, “Cluster 4: IV Ferric Carboxymaltose Therapy” and “Cluster 9: IV Iron Sucrose Treatment” show a significantly higher proportion of pharmaceutical industry funding.

## Discussion

This bibliometric analysis shows that there was exponential publication growth on maternal anaemia, increasing nearly ten-fold from 12 publications in 2000 to a peak of 110 in 2024. Whilst this demonstrates intensified academic attention to maternal anaemia as a priority area, globally there has been insufficient progress in reducing anaemia prevalence in the last decade (3). This disconnect between research output and population-level impact may reflect several challenges, including potential gaps in translating evidence into guidance, barriers to implementing existing recommendations, or insufficient research focused on implementation strategies and real-world programme delivery in high-burden settings (11, 12).

Understanding the extent and distribution of anaemia and contextualising it to specific settings is essential to accelerate action on anaemia. The 2021 Global Burden of Disease found that there were large variations in anaemia burden by age, sex, and geography, with women, and countries in Sub-Saharan Africa and South Asia being particularly affected (13). The bibliometric analysis revealed pronounced geographic imbalances contradicting global disease burden patterns. High-income countries dominated research output with 68.5% of publications (United States, 41.7%; United Kingdom, 26.8%), despite lower anaemia prevalence compared to affected regions. Regional research priorities demonstrated significant specialisation patterns that may not align with local health needs.

To facilitate progress on maternal anaemia, WHO's *Accelerating anaemia reduction: a comprehensive framework for action* emphasizes a broad approach to diagnosis, prevention and management (3). This includes addressing the direct causes, such as micronutrient deficiencies, infectious diseases, gynaecological and obstetric conditions and inherited red blood cell disorders, along with its intermediate and underlying risk factors, such as food insecurity, poor access to health and nutrition services and family planning, and poverty. Whilst the current body of literature is dominated by iron-related research, the framework recognizes that maternal anaemia is aetiologically diverse. Research specifically addressing these non-iron causes of maternal anaemia remains limited within the indexed literature, which is a meaningful gap that can be addressed in future research. Effective prevention and management strategies must be tailored to the dominant aetiology in each context.

Although this bibliometric analysis identified research addressing many areas of the WHO Comprehensive Framework, research focused specifically on prevention strategies was less prominent. Our analysis shows that only about 20% of studies addressed anaemia prevention, with research concentrated on iron and folic acid supplementation strategies, particularly in low-income populations, pregnant women living with HIV, and malaria prophylaxis. The limited research on anaemia diagnosis, including appropriate screening approaches, biomarker thresholds and point-of-care tools, carries direct clinical consequences on timely and accurate diagnosis and treatment. Addressing this gap is essential in implementing the WHO recommendation for universal anaemia screening in antenatal care and to extend diagnostic practice beyond haemoglobin measurement to include cause-specific assessment.

Clarity is needed on optimal methods for anaemia screening, iron supplementation dosing regimens, and the appropriate timing, duration and route of administration in pregnancy. The WHO 2016 recommendations on antenatal care for a positive pregnancy experience identified priority research questions related to iron supplementation (14). However, the bibliometric analysis found that research on iron status and supplementation demonstrated the most pronounced decline among all topics from 2016 to 2024. On the other hand, ferric carboxymaltose, an intravenous iron therapy, exhibited the most pronounced growth trajectory. Research comparing the administration of oral vs. intravenous iron demonstrated the second-highest growth rate. It is important to note that growth in publications on ferric carboxymaltose reflects increasing research interest in this modality and should not be interpreted as evidence of clinical superiority over other iron preparations; establishing comparative clinical effectiveness requires direct head-to-head trials and systematic reviews, which are outside the scope of this bibliometric analysis.

Postpartum haemorrhage and anaemia share a bidirectional relationship: pre-existing anaemia increases a woman's vulnerability to the consequences of blood loss, whilst postpartum haemorrhage is itself one of the leading precipitants of postpartum anaemia. Whilst this analysis did not quantify publications that explicitly examine this dynamic, the prominence of postpartum haemorrhage-related keywords across Clusters 2, 3 and 4 suggests that this intersection is an active area of research, albeit rarely framed in explicitly bidirectional terms. Future synthesis work would benefit from examining this relationship as a discrete focus.

Our results show that the field has evolved towards treatment-focused and outcome evaluation research with little on prevention, diagnosis and implementation. Whilst sustained attention to these areas is needed, new evidence on health outcomes is valuable for informing treatment recommendations on maternal anaemia. Effective anaemia programming requires evidence-based, data-driven, contextualised multisectoral strategies with coordinated implementation. A lack of implementation research hinders the effective scaling of anaemia interventions, particularly in low-resource settings. Limited implementation research means less is known about barriers to intervention delivery and uptake in real-world settings.

As the causes of anaemia are multifactorial, WHO's Comprehensive Framework advocates for coordinated action across systems and multisectoral approaches that address both biological and social determinants. To date, there has been limited research on integrated delivery of anaemia interventions, despite these recommendations and its complex aetiology. Our analysis found that there is a lack of interdisciplinary research. The majority of papers are situated within the field of obstetrics and gynaecology. Public health accounted for about 10% of the papers with contributions increasing markedly after 2010. Other disciplines included general and internal medicine (14.9%), haematology (8.9%), and nutrition & dietetics (8.4%). Whilst the analysis did not specifically examine collaboration between disciplines, it examined the relationship between topic clusters points to opportunities for interdisciplinary research that currently remain largely unrealised.

The research gaps identified in this analysis carry direct implications for future research prioritisation and policy development. The relative underrepresentation of prevention, diagnosis and implementation research, particularly from high-burden regions, reflects a misalignment between current research investment and global health need. Funders and research institutions should consider prioritising implementation science studies that examine how evidence-based interventions can be delivered effectively at scale in low-resource settings. Policy bodies, including WHO and national ministries of health, can use these findings to identify where guideline development is currently impeded by insufficient evidence. Research on integrated, multisectoral approaches to anaemia reduction is a gap requires attention to both a discipline-specific research model and deliberate cross-sector investment.

### Study strengths and limitations

This study demonstrates several methodological strengths that enhance the validity and comprehensiveness of findings. The integration of multiple analytical approaches, including traditional bibliometric indicators and advanced topic modelling using LDA provides triangulated evidence strengthening interpretive confidence. This methodological pluralism aligns with best practices in bibliometric research advocated by Donthu et al. (2021) (15), enabling comprehensive examination of research landscape complexity beyond single-method limitations.

The substantial dataset encompassing 1,007 articles across a 25-year timespan (2000–2024) provides a robust temporal perspective for identifying meaningful trends and evolution patterns. This longitudinal scope enables detection of significant changes in research priorities whilst maintaining a sufficient sample size for statistical trend analysis. The global coverage, spanning 84 countries and 688 institutions, offers comprehensive geographic representation, facilitating identification of research concentration patterns and geographic disparities that would remain invisible in smaller or regionally focused analyses.

The study's examination of research-burden inversion through geographic analysis addresses critical equity considerations often neglected in bibliometric research. By systematically comparing research output concentration with global disease burden patterns, this analysis provides evidence for research justice arguments and highlights structural inequities in knowledge production systems.

The bibliometric approach cannot assess research quality, methodological rigour, or practical impact beyond citation metrics, which exhibit known limitations including citation bias towards certain study types and geographic regions (16). Reliance on Web of Science Core Collection as the primary data source, introduces potential selection bias as this database exhibits known coverage limitations for publications from low- and middle-income countries and non-English language journals (Mongeon & Paul-Hus, 2016) (17). The restriction to English-language publications introduces language bias that may exclude relevant research published in local languages, particularly in regions with high anaemia burden where local language publishing traditions remain strong. These limitations may have resulted in underrepresentation of research conducted in high-burden regions.

The LDA topic modelling approach, whilst providing objective thematic clustering, inherently reduces complex research content to word co-occurrence patterns, potentially obscuring nuanced conceptual relationships and contextual meaning. As noted by Blei (2012) (18), topic models represent statistical abstractions that may not fully capture semantic complexity or disciplinary knowledge structures. The manual assignment of topic labels introduces subjective interpretation and may not reflect universal consensus on thematic boundaries.

## Conclusion

This bibliometric analysis reveals substantial growth in maternal anaemia research over 25 years, with publications increasing nearly ten-fold. However, the research landscape shows thematic imbalances that does not align with global health priorities and disease burden distribution. Thematically, the field has evolved towards treatment-focused and outcome evaluation research while there is little on prevention, diagnosis and implementation. This provides an opportunity to capitalize on new evidence to develop recommendations on treatment strategies. However, sustained attention is needed on prevention and diagnosis for the generation of guidance on these domains, but also to support integrated guidance for treatment and outcomes.

## Data Availability

The original contributions presented in the study are included in the article/Supplementary Material, further inquiries can be directed to the corresponding author.
