# Supplementary material for: Mapping the landscape of anaemia research in pregnant and postpartum women: a bibliometric analysis & topic modelling study
Source: Front Glob Womens Health. 2026 Jul 24;7:1851276. doi: 10.3389/fgwh.2026.1851276 (PMC13447317; doi:10.3389/fgwh.2026.1851276)
Supplement: Supplementary file 1 [file Datasheet1.docx]

**Annexes**

**eFigure 1: Authors’ collaborations network**

**eFigure 2: Institutions’ collaborations network**

**eFigure 3: Countries’ collaborations network**

**Appendix 1 METHODS**

**Dataset Collection (Step 1)**

*Database Selection and Search Strategy:* The Web of Science Core Collection served as the sole bibliographic database for this analysis, selected for its comprehensive coverage of high-impact journals and robust metadata quality essential for advanced bibliometric analysis. A systematic four-phase search strategy was implemented to ensure comprehensive yet focused dataset construction.

Phase 1: Initial Broad Search commenced with an extensive query designed to capture all potentially relevant literature on maternal anaemia. The search strategy employed a combination of Medical Subject Headings (MeSH) terms and free-text keywords, including variations of "anaemia," "anemia," "iron deficiency," combined with pregnancy-related terms such as "pregnant," "pregnancy," "postpartum," "maternal," and "perinatal." This initial search yielded 18,768 citations spanning the complete temporal range from 2000 to 2024.

Phase 2: Query Refinement involved systematic narrowing of search parameters through exclusion criteria designed to eliminate studies unrelated to maternal anaemia in pregnancy. Exclusion terms included "chemotherapy," "oncology," "malignancy," "surgery," and other clinical contexts not relevant to maternal anaemia in pregnancy. This refinement process reduced the dataset to 1,529 citations.

Phase 3: Manual Screening and Selection comprised comprehensive title and abstract screening conducted by reviewers using predefined inclusion and exclusion criteria. Inclusion criteria required studies to focus specifically on anaemia in pregnant or postpartum women, including epidemiological investigations, intervention studies, diagnostic research, and outcome assessments. Exclusion criteria eliminated studies addressing anaemia in non-pregnant populations, paediatric anaemia unrelated to maternal status, and anaemia secondary to non-obstetric causes. This screening process resulted in 1,057 included studies, representing a 69.1% inclusion rate from the refined search results.

Phase 4: WHO Framework Classification involved systematic manual labelling of included studies according to the six WHO Framework categories: Determinants (risk factors and causation), Diagnosis (biomarkers and diagnostic approaches), Prevention (supplementation and preventive interventions), Treatment (therapeutic management), Outcomes (maternal and neonatal health consequences), and Implementation (health systems and policy integration). Multiple reviewers independently classified each study, with disagreements resolved through consensus discussion. Additional 50 studies were excluded at this stage.

**Dataset Pre-processing and Refinement (Step 2)**

*Text Corpus Preparation:* Comprehensive bibliographic data extraction captured titles, abstracts, author keywords, Keywords Plus, publication years, author affiliations, and citation information for all included studies. Text preprocessing employed natural language processing techniques including stop word removal using an expanded English stop word list supplemented with domain-specific terms (e.g., "study," "research," "analysis"). Tokenisation decomposed text into individual terms, whilst lemmatisation reduced words to their root forms to ensure semantic consistency across variant spellings and grammatical forms.

*Keywords Corpus Generation:* A comprehensive vocabulary corpus was constructed from author keywords, Keywords Plus terms, and frequently occurring terms extracted from titles and abstracts. Term frequency analysis identified dominant concepts, whilst rare terms (appearing in fewer than five articles) were excluded to reduce noise. The resulting keywords corpus served as the foundation for semantic network analysis and topic model vocabulary construction.

*Refinement and Normalisation:* Textual data underwent standardisation procedures including acronym expansion, British-American spelling normalisation, and medical terminology standardisation to ensure consistency across the corpus. Author affiliation data were cleaned and standardised to enable accurate institutional and country-level analysis, with institutional name variants consolidated under primary institutional identifiers.

*Weighting Scheme:* Term frequency-inverse document frequency (TF-IDF) weighting was applied to enhance the significance of terms that appear frequently within individual articles but relatively rarely across the entire corpus. This weighting scheme improved the specificity of topic modelling by emphasising distinctive rather than common vocabulary, thereby enhancing the interpretability of extracted topics and reducing the influence of generic academic language.

**Data Analysis (Step 3)**

*Descriptive Analysis:* Publication frequency analysis calculated annual publication counts, cumulative growth patterns, and exponential growth modelling using least-squares regression. Geographic distribution assessment examined publication counts by country and region, normalised by population and gross domestic product to identify research intensity patterns. Institutional productivity analysis ranked universities and research centres by publication output whilst controlling for institution size and research capacity. Journal analysis assessed publication venues, impact factors, and disciplinary distribution to characterise the academic landscape surrounding maternal anaemia research.

*Bibliometric Network Analysis:* Co-authorship Network Construction employed VOSviewer software to visualise collaborative relationships among authors, institutions, and countries. Network parameters included minimum publication thresholds (authors: 3 publications; institutions: 5 publications; countries: 10 publications) to ensure network clarity whilst maintaining comprehensive coverage. Centrality measures (degree, betweenness, closeness) identified influential nodes and network bridges facilitating knowledge transfer.

Institutional and Country Collaboration Networks mapped international research partnerships and institutional linkages, weighted by collaboration frequency and adjusted for geographic distance to identify preferential collaboration patterns. Network clustering algorithms detected research communities and disciplinary boundaries within the maternal anaemia research landscape.

*Topic Modelling:* Latent Dirichlet Allocation Implementation utilised the gensim Python library to identify latent thematic structures within the text corpus. Document preprocessing included additional medical terminology normalisation and phrase detection to capture multi-word concepts (e.g., "postpartum haemorrhage," "iron deficiency"). The LDA algorithm assumed each article contained multiple topics in varying proportions, enabling nuanced thematic classification.

Optimal Topic Number Determination employed systematic evaluation of topic coherence scores and model perplexity across topic numbers ranging from 5 to 15. Coherence scores measured the semantic consistency of topic-defining words, whilst perplexity assessed model fit to the data. The optimal number of topics was selected based on coherence score maximisation and human interpretability assessment by domain experts.

Temporal Topic Analysis tracked topic percentage of articles across the 25-year study period using document-topic probability distributions aggregated by publication year (**Annex 1**). Linear regression analysis identified topics with statistically significant increasing or decreasing trends, whilst change-point detection algorithms identified periods of rapid thematic shifts. Topic evolution visualisation employed stream graphs to illustrate temporal dynamics and research focus transitions.

**Gap Analysis Framework**

*Two-Dimensional Thematic Mapping:* The gap analysis framework employed a two-tiered strategy combining computational topic detection with expert-informed WHO Framework classification. Computer-generated LDA topics were systematically mapped onto WHO Framework categories through content analysis of topic-defining keywords and representative documents. This mapping process identified areas of topic overlap, WHO Framework categories lacking corresponding computational topics, and computational topics spanning multiple WHO Framework domains.

**Trend Analyses Methodology Summary**

**A: Methodology**

**B: Possible Scenarios**

***Regional and Temporal Pattern Assessment:*** Cross-Regional Comparison Analysis calculated topic and WHO Framework category percentage of articles within seven global regions: Sub-Saharan Africa, North Africa & Middle East, South Asia, East Asia & Pacific, Europe & Central Asia, Latin America & Caribbean, and North America. Regional research intensity was normalised by population and anaemia burden estimates to identify research gaps relative to health need.

Linear Regression Trend Analysis assessed directional changes in research focus through temporal regression analysis of topic and WHO Framework category percentage of articles . Slope coefficients quantified annual percentage change rates, whilst statistical significance testing (p < 0.05) identified meaningful trends versus random fluctuations. Trend analysis employed robust regression techniques to minimise the influence of outlier years and publication irregularities.

Research Priority Evolution Assessment examined shifts in research emphasis by comparing early period (2000-2005) and recent period (2020-2024) topic distributions. This temporal comparison identified emerging research areas, declining research domains, and stable research focuses, enabling projection of future research trajectory and identification of sustained versus transient research priorities.

**Interactive Dashboard Development**

The dashboard architecture incorporated multiple interconnected modules enabling users to explore different dimensions of the maternal anaemia in pregnancy research landscape. Data preprocessing for dashboard integration involved standardisation of all analytical outputs into compatible formats, including network data serialisation, topic model parameter storage, and temporal trend data structuring. Interactive visualisation libraries including Plotly, NetworkX, and custom JavaScript components were integrated to enable dynamic data exploration with real-time filtering and drill-down capabilities.

**Data exploration dashboard screenshot**

**APPENDIX 2**

**Authorship and Collaboration Patterns**

The research landscape demonstrated substantial collaborative engagement, with 967 multi-authored articles (96.0%) compared to 40 single-authored publications (4.0%). A total of 5,222 authors contributed to the corpus, yielding an average of 1.16 articles per author and an average collaboration index of 5.82. Author-generated keywords numbered 1,728, whilst Keywords Plus totalled 1,441. Institutional productivity averaged 8.62 articles per institution, and source productivity averaged 2.77 articles per journal or publication venue.

**Author Productivity and Collaboration Networks**

Individual author productivity analysis identified ten researchers with the highest publication output in maternal anaemia research (**eFigure 1**). Breymann, Christian emerged as the most prolific author with 12 publications, followed by Bodnar, LM with 10 publications. Dewey, Kathryn G. contributed 9 publications, whilst Milman, Nils and Roberts, Ian each authored 8 publications. Five researchers shared equal productivity with 7 publications each: Cogswell, ME; Sheiner, Eyal; Seid, Melvin H.; Knight, Marian; and Nair, Manisha.

The author collaboration network visualisation revealed distinct research clusters and collaborative partnerships. The network demonstrated a core-periphery structure with Breymann, Christian positioned centrally, indicating extensive collaborative relationships. Notable collaboration clusters included partnerships between Dewey, Kathryn G. and associated researchers, and a separate cluster centred around Nair, Manisha and Knight, Marian. The network displayed moderate density with 38 nodes and multiple interconnected pathways, suggesting active cross-institutional collaboration within the maternal anaemia research community.

**Institutional Distribution and Networks**

Institutional productivity analysis revealed concentrated research output among leading academic centres (**eFigure 2**). The University of Toronto demonstrated the highest institutional productivity with 58 publications, followed closely by Harvard University with 52 publications. The University of London contributed 40 publications, whilst Université Paris Cité and the University of North Carolina Chapel Hill each produced 39 publications. The University of California System generated 38 publications, and the Harvard T.H. Chan School of Public Health and Assistance Publique Hôpitaux Paris (APHP) each contributed 33 publications. The London School of Hygiene & Tropical Medicine and the University of North Carolina completed the top ten with 32 publications each.

The institutional collaboration network demonstrated extensive international partnerships and research connectivity. The network revealed three primary institutional clusters: a North American cluster centred around Harvard University, University of Toronto, and Johns Hopkins University; a European cluster featuring the London School of Hygiene & Tropical Medicine and associated UK institutions; and an Asia-Pacific cluster including institutions from India, Australia, and other regional partners. The network structure indicated 46 nodes with substantial interconnectivity, reflecting robust international collaboration in maternal anaemia research.

**APPENDIX 3 Geographic Distribution and Regional Research Patterns**

High-Focus Regional Specialisations

Latin America & Caribbean demonstrated the most pronounced regional specialisation patterns, exhibiting exceptionally high focus on specific topics. Iron Status and Supplementation dominated regional research with 85.7% percentage of articles , representing the highest topic concentration observed across all regions. Maternal-Fetal Outcomes similarly achieved 85.7% regional percentage of articles , whilst Iron & Folic Supplementation and Oral vs. IV Iron both maintained substantial regional focus at 78.6% percentage of articles each. This pattern indicated strong regional emphasis on preventive and comparative therapeutic research approaches.

East Asia & Pacific showed concentrated research attention on postpartum complications, with Postpartum Risk Factors and Depression achieving 75.0% regional percentage of articles . Maternal-Fetal Outcomes reached 57.4% percentage of articles , whilst Severe Maternal Morbidity and Mortality maintained 51.5% focus. The region demonstrated balanced attention across outcome-focused research domains.

South Asia exhibited concentrated focus on severe maternal complications, with Severe Maternal Morbidity and Mortality achieving 71.8% regional percentage of articles . Maternal-Fetal Outcomes maintained 50.3% percentage of articles , whilst Postpartum Risk Factors and Depression reached 48.6% regional focus, indicating priority attention to acute maternal health outcomes.

Moderate Regional Concentrations

High-Income Countries demonstrated relatively balanced topic distribution with moderate concentrations across multiple domains. Severe Maternal Morbidity and Mortality achieved 49.5% percentage of articles , whilst Postpartum Risk Factors and Depression and Ferric Carboxymaltose Therapy both maintained 48.0% and 48.4% percentage of articles respectively. This pattern suggested diversified research portfolios with broad thematic coverage.

Sub-Saharan Africa showed mixed research priorities with Severe Maternal Morbidity and Mortality achieving 51.7% percentage of articles and Postpartum Risk Factors and Depression reaching 57.3%. However, the region demonstrated substantial variation across other topics, with some showing moderate focus and others exhibiting significant gaps.

Pronounced Regional Research Gaps

Limited Research Focus Areas: Several regions demonstrated markedly low research attention in specific domains, indicating potential research gaps. Sub-Saharan Africa exhibited minimal focus on IV Iron Sucrose Treatment (7.9%) and Oral vs. IV Iron (16.9%), suggesting limited attention to advanced therapeutic comparisons despite high anaemia burden. East Asia & Pacific showed restricted focus on IV Iron Sucrose Treatment (7.4%) and Hematologic Disorders Diagnosis (11.8%).

North Africa & Middle East demonstrated the most pronounced research gaps across multiple domains, with IV Iron Sucrose Treatment achieving only 12.5% percentage of articles and Oral vs. IV Iron reaching 25.0%. Hematologic Disorders Diagnosis maintained 29.2% percentage of articles , whilst Iron Status and Supplementation achieved only 29.2% regional focus, indicating substantial research underrepresentation across key domains.

Latin America & Caribbean exhibited selective research gaps despite high overall activity, with IV Iron Sucrose Treatment achieving only 21.4% percentage of articles and Hematologic Disorders Diagnosis reaching 28.6%, suggesting limited attention to specific diagnostic and advanced therapeutic domains.

Regional Research Intensity Patterns

High-Intensity Regions: Latin America & Caribbean demonstrated the highest research intensity with multiple topics exceeding 70% percentage of articles , followed by East Asia & Pacific with several topics above 50% percentage of articles . These regions showed concentrated research efforts with clear thematic priorities.

Moderate-Intensity Regions: High-Income Countries and South Asia exhibited moderate research intensity with balanced topic distribution, suggesting diversified research approaches without extreme specialisation patterns.

Low-Intensity Regions: North Africa & Middle East and Other/Unknown regions demonstrated lower overall research intensity with few topics exceeding 45% percentage of articles , indicating potential underrepresentation in maternal anaemia research or limited research capacity in specific thematic domains.

The geographic analysis revealed significant research inequities, with some regions demonstrating concentrated expertise in specific domains whilst exhibiting substantial gaps in others, highlighting opportunities for enhanced international collaboration and capacity building in underrepresented areas.

**APPENDIX 4 Nine-Cluster Thematic Structure**

**Cluster Analysis Summary**

| ***Cluster*** | **Details** |
| --- | --- |
| ***Cluster 1: Iron Status and Supplementation*** | **Total Articles:** 150 (14.9%) **Key Themes:** Iron status, serum ferritin, vitamin supplementation, infant health **Notable Articles:** - Duggan et al. (2014): Vitamin B-12 supplementation enhances maternal and infant vitamin B-12 status. - Beard et al. (2005): Maternal iron deficiency anemia affects postpartum emotional and cognitive health. **Summary:** Focuses on iron and nutritional interventions in pregnancy/postpartum, highlighting impacts on maternal and infant health. |
| ***Cluster 2: Postpartum Risk Factors and Depression*** | **Total Articles:** 250 (24.8%) **Key Themes:** Postpartum hemorrhage risk, depression, cesarean section outcomes **Notable Articles:** - Zeng et al. (2024): Risk prediction model for postpartum hemorrhage after cesarean. - Anjum & Batool (2019): Factors contributing to postpartum depression in Pakistan. **Summary:** Examines risk factors for postpartum complications, emphasizing predictive models and interventions. |
| ***Cluster 3: Severe Maternal Morbidity and Mortality*** | **Total Articles:** 326 (32.4%) **Key Themes:** Maternal mortality, morbidity, blood transfusion, cesarean complications **Notable Articles:** - Swarupa et al. (2018): Heart disease-related maternal deaths. - Siddiqui et al. (2012): Severe obstetric morbidity outcomes in Karachi. **Summary:** Analyzes severe obstetric outcomes, including near-miss events and care quality. |
| ***Cluster 4: Ferric Carboxymaltose and Anemia Management*** | **Total Articles:** 87 (8.6%) **Key Themes:** Intravenous ferric carboxymaltose, anemia management, blood loss reduction **Notable Articles:** - Toblli & Angerosa (2014): Ferric carboxymaltose as optimized iron delivery. - Scott (2018): Evidence for ferric carboxymaltose effectiveness. **Summary:** Highlights clinical evidence for ferric carboxymaltose in pregnancy/postpartum anemia. |
| ***Cluster 5: Thrombocytopenia and Postpartum Complications*** | **Total Articles:** 63 (6.3%) **Key Themes:** Thrombocytopenic conditions, puerperal sepsis, anemia awareness **Notable Articles:** - Baghli et al. (2018): Atypical hemolytic uremic syndrome postpartum. - Krishnan & Kirubamani (2019): Anemia awareness gaps in pregnancy. **Summary:** Focuses on thrombocytopenia-related complications and public health awareness. |
| ***Cluster 6: Maternal-Fetal Outcomes and Complications*** | **Total Articles:** 203 (20.2%) **Key Themes:** Fetal outcomes, neonatal health, thyroid dysfunction **Notable Articles:** - Nazarpour et al. (2015): Thyroid dysfunction impacts on pregnancy. - Cakmak et al. (2018): First-trimester hemoglobin effects on outcomes. **Summary:** Explores maternal-fetal health, including thyroid dysfunction and hemoglobin effects. |
| ***Cluster 7: Intravenous vs. Oral Iron Therapy*** | **Total Articles:** 71 (7.1%) **Key Themes:** IV iron, oral iron, postpartum anemia, therapeutic effectiveness **Notable Articles:** - Breymann et al. (2010): IV vs. oral iron for postpartum anemia. - Lower et al. (2024): Standardized protocols for postpartum anemia. **Summary:** Compares IV and oral iron treatments, focusing on efficacy and implementation. |
| ***Cluster 8: Iron and Folic Acid Supplementation*** | **Total Articles:** 59 (5.9%) **Key Themes:** Supplementation efficacy, malaria prophylaxis, public health **Notable Articles:** - Mitra & Khoury (2012): Universal iron supplementation strategies. - Finkelstein et al. (2012): Anemia predictors in HIV-infected pregnant women. **Summary:** Evaluates supplementation programs in low-income/HIV-affected populations. |
| ***Cluster 9: Iron Sucrose Treatment Efficacy*** | **Total Articles:** 59 (5.9%) **Key Themes:** IV iron sucrose, third-trimester anemia, postpartum deficiency **Notable Articles:** - Bhandal & Russell (2006): IV iron sucrose vs. oral iron postpartum. - Krafft & Breymann (2011): Erythropoietin adjuvant with iron sucrose. **Summary:** Clinical studies on iron sucrose for postpartum anemia treatment. |

Network Structure and Co-occurrence Patterns

The topic relationship network revealed complex interdisciplinary relationships among the nine research domains identified through LDA topic modelling, with varying degrees of connectivity and collaborative potential. Network analysis identified three distinct categories based on connectivity patterns: Central Topics (red nodes), Bridging Topics (orange nodes), and Specialised Topics (blue nodes). Node sizes corresponded to topic frequency within the corpus, whilst edge thickness represented co-occurrence strength between topics, with numerical values indicating the number of studies exhibiting dual topic presence.

**Figure 5: Topic Relationship Network**

Central Topics: IV Iron Sucrose Treatment emerged as the primary central node with the highest connectivity across multiple research domains (64 studies total frequency). This topic demonstrated substantial co-occurrence with Ferric Carboxymaltose Therapy (10 studies), Oral vs. IV Iron (10 studies), and Iron Status and Supplementation (14 studies), indicating its fundamental role in connecting diverse therapeutic approaches within maternal anaemia literature. The central positioning reflected IV Iron Sucrose Treatment's integration with both comparative effectiveness research and broader supplementation strategies. Oral vs. IV Iron functioned as another central topic (53 studies), serving as a critical junction connecting treatment modalities with prevention approaches. This topic demonstrated moderate co-occurrence with Hematologic Disorders Diagnosis (4 studies) and Iron & Folic Supplementation (7 studies), suggesting its role in bridging therapeutic decision-making with diagnostic considerations and preventive interventions.

Bridging Topics: Ferric Carboxymaltose Therapy served as the primary bridging topic (59 studies), facilitating connections between advanced therapeutic approaches and broader research domains. Beyond its strong connection with IV Iron Sucrose Treatment (10 studies), this topic demonstrated significant co-occurrence with Postpartum Risk Factors and Depression (40 studies), indicating integration of advanced iron therapy research with maternal mental health and complication assessment. Hematologic Disorders Diagnosis functioned as a crucial bridging topic (31 studies), connecting diagnostic research with multiple clinical domains. This topic demonstrated connections with Maternal-Fetal Outcomes (9 studies), Severe Maternal Morbidity and Mortality (16 studies), and moderate linkage with Oral vs. IV Iron (4 studies), suggesting its role in linking diagnostic approaches with outcome assessment and treatment selection. Iron Status and Supplementation represented another significant bridging topic (128 studies), connecting prevention-focused research with treatment approaches. This topic demonstrated strong co-occurrence with IV Iron Sucrose Treatment (14 studies) and substantial connection with Iron & Folic Supplementation (40 studies), indicating its role in linking biomarker assessment with therapeutic interventions.

Specialised Topics: Severe Maternal Morbidity and Mortality exhibited selective connectivity patterns (217 studies), maintaining substantial connections with Postpartum Risk Factors and Depression (62 studies) and moderate linkage with Hematologic Disorders Diagnosis (16 studies). This pattern indicated the topic's specialised focus on acute maternal complications whilst maintaining selective integration with risk assessment and diagnostic research.

Maternal-Fetal Outcomes demonstrated limited connectivity (176 studies) with primary connection to Hematologic Disorders Diagnosis (9 studies) and weak linkage to Severe Maternal Morbidity and Mortality (54 studies). This specialisation suggested focused research attention on pregnancy outcomes with minimal integration across other research domains. Postpartum Risk Factors and Depression showed concentrated connectivity (227 studies) primarily linking with Severe Maternal Morbidity and Mortality (62 studies) and Ferric Carboxymaltose Therapy (40 studies), indicating specialised focus on postpartum complications with selective therapeutic integration.

Co-occurrence Strength Analysis

High-Strength Connections: The Postpartum Risk Factors and Depression relationship with Severe Maternal Morbidity and Mortality demonstrated the strongest co-occurrence (62 studies), indicating substantial research integration between postpartum complications and severe maternal outcomes. The connection between Severe Maternal Morbidity and Mortality and Maternal-Fetal Outcomes (54 studies) represented another high-strength association, suggesting integrated approaches to maternal and fetal health assessment.

Moderate-Strength Connections: The Iron Status and Supplementation relationship with Iron & Folic Supplementation (40 studies) and Ferric Carboxymaltose Therapy connection with Postpartum Risk Factors and Depression (40 studies) demonstrated moderate co-occurrence, indicating balanced research attention spanning prevention, treatment, and outcome domains.

Emerging Therapeutic Clusters: The treatment-focused cluster comprising IV Iron Sucrose Treatment, Ferric Carboxymaltose Therapy, and Oral vs. IV Iron showed interconnected relationships (10 studies each between primary nodes), suggesting emergence of comparative effectiveness research as a distinct domain within maternal anaemia literature.

**Temporal Trend Analysis of Topic Categories**

**Figure 6: Trend Analysis of Topic Categories**

Linear regression analysis of topic percentage of articles over the 25-year study period identified significant temporal trends across the nine thematic clusters (**Figure 6**). Trend analysis revealed varying patterns of research evolution, with slope coefficients ranging from +0.156 to -0.267, indicating substantial shifts in research focus and priorities within maternal anaemia literature.

**APPENDIX 5 WHO Framework Classification**

| s/n | Category | Number | Percentage |
| --- | --- | --- | --- |
| **1** | **Risk factors** | **380** | **3.74** |
| 1a | Biological mechanisms (blood loss, haemolysis, ineffective erythropoiesis) | 69 | 6.85 |
| 1b | Direct causes (micronutrient deficiencies; infectious and chronic diseases; gynecological and obstetric conditions, eg PPH; inherited red blood cell disorders) | 264 | 26.22 |
| 1c | Intermediate risk factors (food insecurity, poor diet, lack of access to and use of family planning, limited access to health and nutrition services, inadequate access to WaSH) | 75 | 7.45 |
| 1d | Underlying risk factors (low education, poverty, conflict, cultural norm and behaviors, health policies, socio-demographics, politics, economy, inequity) | 131 | 13.01 |
| **2** | **Diagnosis** | **153** | **15.19** |
| 2a | Biomarkers, lab assessment & QA | 102 | 10.13 |
| 2b | Physical signs & symptoms | 10 | 0.99 |
| 2c | Screening approaches & protocols | 22 | 2.18 |
| 2d | Cause-specific diagnosis (incl. ferritin) | 33 | 3.28 |
| 2e | Point-of-care & innovative diagnostics | 4 | 0.4 |
| 2f | Diagnostic algorithms & decision support | 23 | 2.28 |
| **3** | **Prevention** | **82** | **8.14** |
| 3a | Nutrition (diet, fortification, supplementation) | 64 | 6.36 |
| 3b | Infectious diseases (bednets, deworming, IPTp, WaSH) | 7 | 0.7 |
| 3c | Family planning | 2 | 0.2 |
| 3d | Multi-component interventions | 2 | 0.2 |
| 3e | Education & counselling | 3 | 0.3 |
| 3f | Intervention adherence & compliance | 5 | 0.5 |
| 3g | Management of side effects | 2 | 0.2 |
| **4** | **Treatment** | **290** | **28.80** |
| 4a | Oral micronutrient supplementation | 115 | 11.42 |
| 4b | Intravenous iron | 129 | 12.81 |
| 4c | Blood transfusion | 87 | 8.64 |
| 4d | Treatment of infections (malaria, etc) | 1 | 0.1 |
| 4e | Multi-component interventions | 51 | 5.06 |
| 4f | Treatment adherence & compliance | 6 | 0.6 |
| 4g | Management of side effects | 9 | 0.89 |
| 4h | Treatment monitoring & response | 18 | 1.79 |
| **5** | **Outcomes** | **314** | **31.18** |
| 5a | Impact assessment | 314 | 31.18 |
| **6** | **Implementation** | **14** | **1.39** |
| 6a | Integration in health care systems | 4 | 0.4 |
| 6b | Implementation strategies | 3 | 0.3 |
| 6c | Access & coverage of interventions/services | 9 | 0.89 |

**The Evolving Landscape of Maternal Anaemia Research Funding**

**Top most cited funding source:**

| Source | Count |
| --- | --- |
| Bill & Melinda Gates Foundation | 48 |
| NIH | 25 |
| MRC UKRI | 16 |
| National Institutes of Health Research (NIHR) | 10 |
| Vifor Pharma | 9 |
| Wellcome Trust | 9 |
| National Natural Science Foundation of China | 8 |
| United States Agency for International Development | 5 |
| Micronutrient Initiative | 3 |
| Indian Council of Medical Research | 3 |
| Landsteiner Foundation for Blood Transfusion Research | 3 |
| PHS HHS Medline | 3 |
| Norwegian Research Fund for General Practice | 2 |

**Profile of Funders: A Diverse Ecosystem**

**Co-Funding Network Analysis**

**Funding Dynamics within Research Clusters**
